# Eosinophil may be a predictor of immune‐related adverse events induced by different immune checkpoint inhibitor types: A retrospective multidisciplinary study

**DOI:** 10.1002/cam4.6724

**Published:** 2023-11-21

**Authors:** Yoshihiko Tasaki, Yosuke Sugiyama, Shuzo Hamamoto, Taku Naiki, Takehiro Uemura, Keisuke Yokota, Daisuke Kawakita, Motoki Nakamura, Ryo Ogawa, Takaya Shimura, Yoshihisa Mimura, Yuji Hotta, Kunihiro Odagiri, Nanami Ito, Moeko Iida, Yuka Kimura, Hirokazu Komatsu, Hiromi Kataoka, Shuji Takiguchi, Akimichi Morita, Shinichi Iwasaki, Katsuhiro Okuda, Akio Niimi, Takahiro Yasui, Yoko Furukawa‐Hibi

**Affiliations:** ^1^ Department of Clinical Pharmaceutics Nagoya City University Graduate School of Medical Sciences Nagoya Aichi Japan; ^2^ Department of Nephro‐Urology Nagoya City University Graduate School of Medical Sciences Nagoya Aichi Japan; ^3^ Department of Respiratory Medicine, Allergy, and Clinical Immunology Nagoya City University Graduate School of Medical Sciences Nagoya Aichi Japan; ^4^ Department of Thoracic and Pediatric Surgery Nagoya City University Graduate School of Medical Sciences Nagoya Aichi Japan; ^5^ Department of Otorhinolaryngology, Head and Neck Surgery Nagoya City University Graduate School of Medical Sciences Nagoya Aichi Japan; ^6^ Department of Geriatric and Environmental Dermatology Nagoya City University Graduate School of Medical Sciences Nagoya Aichi Japan; ^7^ Department of Gastroenterological Surgery Nagoya City University Graduate School of Medical Sciences Nagoya Aichi Japan; ^8^ Department of Gastroenterology and Metabolism Nagoya City University Graduate School of Medical Sciences Nagoya Aichi Japan; ^9^ Department of Hematology and Oncology Nagoya City University Graduate School of Medical Sciences Nagoya Aichi Japan

**Keywords:** biomarker, cancer, eosinophil, immune checkpoint inhibitor, immune‐related adverse event

## Abstract

**Background:**

Immune checkpoint inhibitors (ICIs) can cause severe immune‐related adverse events (irAEs). However, biomarkers for irAEs common to different types of ICIs and cancers have not been reported. This study examined whether eosinophils can be used as a predictor of irAEs.

**Methods:**

Six hundred fourteen patients with cancer (esophageal, gastric, head and neck, lung, melanoma, renal cell, urothelial, and other cancer) received anti‐PD‐1, anti‐PD‐L1, or anti‐CTLA‐4 plus anti‐PD‐1 therapy. The patients were divided into two groups depending on whether they experienced irAEs (irAE group) or not (non‐irAE group). Eosinophils were examined before the two‐course treatment.

**Results:**

Patients in the irAE group who received anti‐PD‐1 or anti‐CTLA‐4 plus anti‐PD‐1 therapy had higher eosinophils before the two‐course treatment than those in the non‐irAE group (*p* < 0.05). The eosinophils in the anti‐PD‐L1 therapy group tended to increase in the irAE group. Furthermore, eosinophils in gastric, head and neck, lung, melanoma, renal, and urothelial cancers were significantly higher in the irAE group than in the non‐irAE group (*p* < 0.05). The optimal cutoff value for eosinophils against irAEs was 3.0% (area under the curve = 0.668). In multivariate analyses, eosinophils of ≥3.0% were an independent factor for irAEs (odds ratio: 2.57, 95% CI: 1.79–3.67).

**Conclusion:**

An increased eosinophil before the two‐course treatment may be a predictor of irAEs in various cancers treated with different ICIs.

## 1 INTRODUCTION

Globally, approximately 19.3 million people are newly diagnosed with cancer, and approximately 10 million die from cancer each year.^1^ Cancer is ranked among the top 10 leading causes of death among people under 70 years of age in most countries.^1^ Therefore, overcoming cancer through treatment is the greatest achievement for humankind. Large‐scale clinical trials have demonstrated that immune checkpoint inhibitors (ICIs), such as anti‐programmed cell death 1 (anti‐PD‐1), anti‐programmed cell death ligand 1 (anti‐PD‐L1), anti‐cytotoxic T‐lymphocyte antigen 4 (anti‐CTLA‐4), play a revolutionary role in improving the prognosis of various cancers.^2^, ^3^, ^4^, ^5^, ^6^, ^7^, ^8^, ^9^, ^10^

ICIs cause immune‐related adverse events (irAEs) in most organ systems by activating the immune system and causing imbalances in immunological tolerance.^11^, ^12^ For instance, approximately 90% of patients with renal cell carcinoma who received anti‐CTLA‐4 plus anti‐PD‐1 combination therapy experienced irAEs of certain grade. In addition, 46% of patients suffered from irAEs of grade ≥3, which are defined as severe and lethal.^13^ Because the detailed mechanisms of irAEs are unclear, it is difficult for physicians to predict when and in what organ systems irAEs occur. Therefore, it is important to identify biomarkers to predict early and control irAEs to a low grade.

The frequency of irAE occurrences is not significantly different across different cancer types, whereas the frequency of grade ≥3 irAEs occurrences is significantly different across different ICI types.^14^ However, several studies on irAE biomarkers have focused on specific ICIs, primary tumors, and irAE type.^12^, ^15^ To date, there are no reports on cross‐sectional biomarkers beyond these types. Recently, it was reported that eosinophils are associated with irAEs in patients who received ICIs.^16^, ^17^, ^18^, ^19^, ^20^ Our previous study showed that increased eosinophil proportion of ≥3.0% is an effective biomarker for predicting irAE occurrences in patients with renal cell carcinoma who received anti‐CTLA‐4 plus anti‐PD‐1 combination therapy.^21^ In this study, we retrospectively investigated whether the proportion of eosinophils in patients with various cancers treated with different ICIs is a predictor of irAE occurrences.

## 2 MATERIALS AND METHODS

### 2.1 Study design and treatment

The 614 patients who received ICIs for the first time, including anti‐PD‐1 (nivolumab [240 or 480 mg/body every 2 or 4 weeks] and pembrolizumab [200 or 400 mg/body every 3 or 6 weeks]), anti‐PD‐L1 (atezolizumab [1200 mg/body every 3 weeks], avelumab [10 mg/kg every 2 weeks] and durvalumab [10 mg/kg every 2 weeks]), anti‐CTLA‐4 (ipilimumab [3 mg/kg every 3 weeks]) monotherapy, or anti‐CTLA‐4 plus anti‐PD‐1 combination therapy (3 mg/kg of ipilimumab and 80 mg/body of nivolumab every 3 weeks, 1 mg/kg of ipilimumab and 240 mg/body of nivolumab every 3 weeks or 1 mg/kg of ipilimumab and 360 mg/body nivolumab every 6 weeks), were enrolled in September 2014 and August 2022 in this retrospective study. Patients who received ICIs in combination with other chemotherapy or molecular targeted agents and those who had eosinophilic disorders were excluded. All patients were followed up until death or loss of contact. To identify a biomarker, we examined the eosinophil proportion and count after blood sampling before one course (baseline sample) and after two courses of treatment with ICIs (two‐course sample). Overall survival (OS) was defined as the period between cancer diagnosis and patient death or last follow‐up. Treatment response was assessed using the Response Evaluation Criteria in Solid Tumors (RECIST) version 1.1.^22^ We defined irAEs as symptoms that suspected to immune dysregulation through the blood sampling and clinical assessment. IrAEs were graded according to the National Cancer Institute Common Terminology Criteria for Adverse Events, version 5.0. Nearest‐neighbor with caliper matching on the propensity score between irAE and non‐irAE groups was used to compensate for differences in age, sex, primary tumor type, immune checkpoint therapy, and use of steroids before ICIs, with the propensity score estimated using a logistic multivariate regression analysis. We compared the patient characteristics, eosinophil proportion and count, OS, and progression‐free survival (PFS) between the matching groups.

### 2.2 Statistical analyses

*p*‐values of statistical significance were set at *p < 0.05. Differences in the quantified data between groups were compared using the *t* test or one‐way analysis of variance (anova) followed by Tukey's test. Fisher's exact test was used to assess differences in patient characteristics. The optimal cutoff points for potential peripheral blood biomarkers to predict irAE onset were determined by analyzing the receiver operating characteristic (ROC) curves. OS and PFS were calculated using the Kaplan–Meier method and log‐rank test. Univariate and multivariate logistic or Cox regression analyses were used to assess the factors associated with irAEs and OS. Statistical analyses were performed using GraphPad Prism 9 software and EZR (Saitama Medical Center, Jichi Medical University).^23^

## 3 RESULTS

### 3.1 Patient characteristics

We classified patients who experienced irAEs into the irAE and non‐irAE groups (Table 1). Of the 614 patients enrolled in this study, 52.8% (*n* = 324) experienced non‐irAEs and 47.2% (*n* = 290) experienced irAEs. In the irAE group, the incidence of primary tumor type was 39.4% (13 of 33) for esophageal cancer, 29.3% (12 of 41) for gastric cancer, 51.8% (44 of 85) for head and neck cancer, 42.4% (64 of 151) for lung cancer, 67.2% (43 of 64) for melanoma, 63.0% (68 of 108) for renal cell carcinoma, 34.2% (40 of 117) for urothelial carcinoma, and 40.0% (6 of 15) for other cancers. The incidence of irAEs in the ICI group was 42.0% (200 of 476) for anti‐PD‐1 monotherapy, 58.3% (28 of 48) for anti‐PD‐L1 monotherapy, and 68.5% (61 of 89) for anti‐CTLA‐4 plus anti‐PD‐1 combination therapy. One percent (6 of 614) had been using steroids continuously prior to ICI therapy.

**TABLE 1:** Clinical features of patients.

Characteristics	Non‐irAE group	irAE group	*p* value
Total, *n* (%)	324 (52.8)	290 (47.2)	
Age (range)	73 (32–94)	74 (41–93)	0.62
Sex, *n* (%)			
Male	228 (50.8)	221 (49.2)	0.12
Female	96 (58.2)	69 (41.8)	
Primary tumor type, *n* (%)			
Esophageal cancer	20 (60.6)	13 (39.4)	0.37
Gastric cancer	29 (70.7)	12 (29.3)	<0.05
Head and neck cancer	41 (48.2)	44 (51.8)	0.41
Lung cancer	87 (57.6)	64 (42.4)	0.18
Melanoma	21 (32.8)	43 (67.2)	<0.05
Renal cell carcinoma	40 (37.0)	68 (63.0)	<0.05
Urothelial carcinoma	77 (65.8)	40 (34.2)	<0.05
Other cancers	9 (60.0)	6 (40.0)	0.61
Colorectal cancer	2	2	
Cancer of unknown primary	1	2	
Mediastinal tumor	1	0	
Merkel cell carcinoma	2	2	
Mesothelioma	3	0	
Immune checkpoint therapy, *n* (%)			
Anti‐PD‐1	276 (58.0)	200 (42.0)	<0.05
Nivolumab	134	119	
Pembrolizumab	142	81	
Anti‐PD‐L1	20 (41.7)	28 (58.3)	0.13
Atezolizumab	11	5	
Avelumab	3	8	
Durvalumab	6	15	
Anti‐CTLA‐4 plus anti‐PD‐1	28 (31.5)	61 (68.5)	<0.05
Anti‐CTLA‐4	0 (0.0)	1 (100)	0.47
Use of steroids before ICIs therapy, *n* (%)	2 (0.6)	4 (1.4)	0.42

Abbreviations: CTLA‐4, cytotoxic T‐lymphocyte antigen 4; ICIs: immune checkpoint inhibitors; irAE, immune‐related adverse event; PD‐1, Programmed death 1; PD‐L1, Programmed death ligand 1.

Age, sex ratio, esophageal cancer, head and neck cancer, lung cancer, other cancers, anti‐PD‐L1 monotherapy, anti‐CTLA‐4 monotherapy, and use of steroids before ICIs did not differ substantially between the irAE and non‐irAE groups (Table 1). The number of patients with melanoma, renal cell carcinoma, and anti‐CTLA‐4 plus anti‐PD‐1 combination therapy in the irAE group was substantially higher than in the non‐irAE group, whereas the number of patients with gastric cancer, urothelial carcinoma, and anti‐PD‐1 monotherapy in the irAE group was markedly lower than in the non‐irAE group (Table 1).

Table S1 presents details of the incidence of irAEs in patients with various cancers treated with each ICIs. Among patients treated with anti‐PD‐1, melanoma showed the highest incidence of irAEs (62.3%, 33 of 53), followed by renal cell carcinoma (54.8%, 17 of 31), head and neck cancer (51.8%, 44 of 85), esophageal cancer (39.4%, 13 of 33), lung cancer (38.9%, 44 of 113), urothelial carcinoma (30.9%, 34 of 110), other cancers (30.0%, 3 of 10), and gastric cancer (29.3%, 12 of 41). The incidence of anti‐PD‐L1 was urothelial carcinoma in 85.7% (6 of 7), lung cancer in 54.1% (20 of 37), and other cancers in 50.0% (2 of 4). Patients with melanoma treated with anti‐CTLA‐4 plus anti‐PD‐1 had a higher incidence of irAEs (90.0%, 9 of 10) than those with renal cell carcinoma (66.2%, 51 of 77).

### 3.2 Frequency of onset to irAEs

Table S2 presents details of the frequency of irAEs. A total of 290 patients experiencing irAEs and 419 events with irAEs of any grade were reported in this study, including 111 events (26.5%) with grade ≥3 irAEs. The incidence of irAEs of any grade for each treatment was 65.9% (*n* = 276) for anti‐PD‐1 monotherapy, 9.6% (*n* = 40) for anti‐PD‐L1 monotherapy, 24.3% (*n* = 102) for anti‐CTLA‐4 plus anti‐PD‐1 combination therapy, and 0.2% (*n* = 1) for anti‐CTLA‐4 monotherapy. The rate of irAEs of grade ≥3 was the highest in anti‐CTLA‐4 plus anti‐PD‐1 combination therapy (40.2%; 41 of 102), followed by anti‐PD‐1 monotherapy (23.6%; 65 of 276), and anti‐PD‐L1 monotherapy (12.5%; 5 of 40). The most common irAEs were skin (29.1%; 122 of 419), endocrine (22.0%; 92 of 419), gastrointestinal (21.7%; 91 of 419), pulmonary (10.7%; 45 of 419), and other (16.5%; 69 of 419).

### 3.3 Increased eosinophil proportion in patients treated with different ICI type reflects irAEs occurrences in a two‐course sample

**FIGURE 1:**
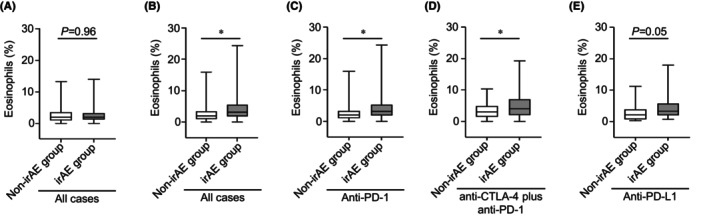
Eosinophil proportion increases before the two‐course treatment with ICIs. (A) Boxplot showing the eosinophil proportion in the baseline sample: non‐irAE (*n* = 319) and irAE (*n* = 288) groups. (B) Boxplot showing the eosinophil proportion in the two‐course sample; non–irAE (*n* = 309) and irAE (*n* = 285) groups. (C–E) Boxplot showing eosinophil proportion in the two‐course sample of patients who received (C) anti‐PD‐1 monotherapy (non‐irAE [*n* = 265] and irAE [*n* = 197] groups), (D) anti‐CTLA‐4 plus anti‐PD‐1 combination therapy (non‐irAE [*n* = 24] and irAE [*n* = 59] groups), and (E) anti‐PD‐L1 monotherapy (non‐irAE [*n* = 20] and irAE [*n* = 28] groups). The median value is represented by the horizontal midline in each box. The bottom and the top of each box are indicated. The 25th and 75th percentiles are represented by the ends of the whiskers, which indicate the minimum and maximum values of all data, respectively. *p < 0.05. (A–E) Unpaired *t* test. irAE, immune‐related adverse event; PD‐1, Programmed death 1; PD‐L1, Programmed death ligand 1.

The proportion of eosinophils in the baseline sample of all patients did not differ between the irAE and non‐irAE groups (mean: 2.5% vs. 2.5%; *p* = 0.96; Figure 1A). Notably, the eosinophil proportion in the two‐course sample of all cases was significantly higher in the irAE group than in the non‐irAE group (mean: 4.3% vs. 2.5%; *p* < 0.05; Figure 1B). Consistent with the results of the eosinophil proportion, the eosinophil count of the irAE group in the two‐course sample was higher than that of the non‐irAE group (Figure S1).

To clarify whether the variation in the eosinophil proportion differed according to the ICI type, we examined this in a two‐course sample. Patients in the irAE group who received anti‐PD‐1 monotherapy (mean: 4.1% vs. 2.4%) and anti‐CTLA‐4 plus anti‐PD‐1 combination therapy (mean: 5.2% vs. 3.4%) showed a higher proportion of eosinophils than those in the non‐irAE group (*p* < 0.05; Figure 1C,D). The proportion of eosinophils in the anti‐PD‐L1 monotherapy group tended to be higher in the irAE group (mean: 4.3% vs. 2.7%; *p* = 0.05; Figure 1E).

### 3.4 Analysis of association between eosinophil proportion and primary tumor type in a two‐course sample

**FIGURE 2:**
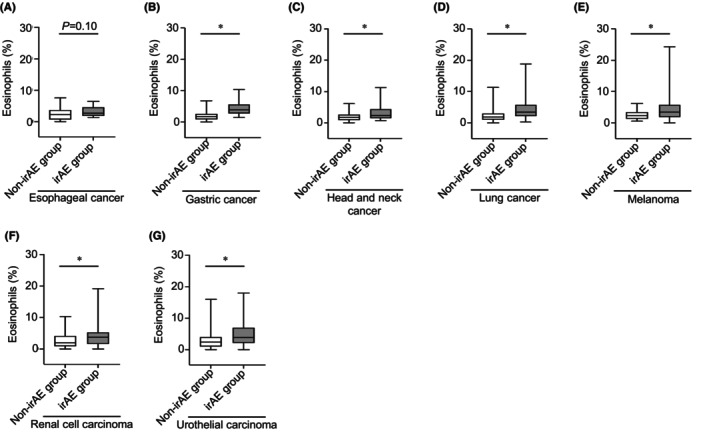
Eosinophil proportion increases in any primary tumor type. (A–G) Boxplot showing eosinophil proportion in the two‐course sample of patients with (A) esophageal cancer (non‐irAE [*n* = 19] and irAE [*n* = 12] groups), (B) Gastric cancer (non‐irAE [*n* = 28] and irAE [*n* = 12] groups), (C) head and neck cancer (non‐irAE [*n* = 41] and irAE [*n* = 43] groups), (D) lung cancer (non‐irAE [*n* = 85] and irAE [*n* = 64] groups), (E) melanoma (non‐irAE [*n* = 20] and irAE [*n* = 41] groups), (F) renal cell carcinoma (non‐irAE [*n* = 35] and irAE [*n* = 67] groups), and (G) urothelial carcinoma (non‐irAE [*n* = 72] and irAE [*n* = 40] groups). The median value is represented by the horizontal midline in each box. The bottom and the top of each box are indicated. The 25th and 75th percentiles are represented by the ends of the whiskers, which indicate the minimum and maximum values of all data, respectively. *p < 0.05. (A–G) unpaired *t* test. IrAEs, immune‐related adverse events.

Differential outcomes of the incidence of irAEs in patients with various cancers treated with each ICIs inspired us to examine whether the proportion of eosinophils in a two‐course sample changes for each primary tumor type. The proportion of eosinophils in esophageal cancer tended to increase in the irAE group in the two‐course sample (mean: 3.2% vs. 2.3%; *p* = 0.10; Figure 2A). Moreover, eosinophil proportion of the irAE group with gastric cancer (mean: 4.5% vs. 2.1%), head and neck cancer (mean: 3.2% vs. 1.9%), lung cancer (mean: 4.5% vs. 2.4%), melanoma (mean: 4.8% vs. 2.5%), renal cell carcinoma (mean: 4.6% vs. 2.7%), and urothelial carcinoma (mean: 5.0% vs. 3.0%) was found to be significantly higher than those of the non‐irAE group in a two‐course sample (*p* < 0.05; Figure 2B–G).

### 3.5 Relationship between the time of irAE occurrences and eosinophil proportion

**FIGURE 3:**
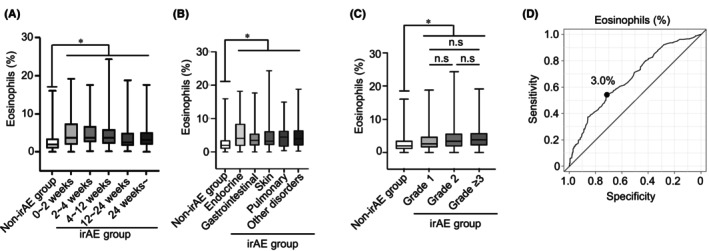
Eosinophil proportion increases, regardless in time of irAE occurrences and irAE type/severity. (A) Box plots showing eosinophil proportion in a two‐course sample of patients who experienced the onset of irAEs after 0–2 weeks (*n* = 44), 2–4 weeks (*n* = 51), 4–12 weeks (*n* = 125), 12–24 weeks (*n* = 62), and 24 weeks (*n* = 72). (B) Plots showing the eosinophil proportion in a two‐course sample of patients who experienced the onset of irAEs (non‐irAE group [*n* = 309], endocrine [*n* = 85], gastrointestinal [*n* = 78], skin [*n* = 108], pulmonary [*n* = 44], and other disorders [*n* = 61]). (C) Boxplots showing eosinophil proportion in a two‐course sample of any graded irAEs (non‐irAE group [*n* = 309], Grade 1 [*n* = 76], Grade 2 [*n* = 112], and Grade ≥3 [*n* = 97]). (D) Receiver operating characteristic curve analysis of eosinophil proportion for the occurrence of irAEs. The median value is represented by the horizontal midline in each box. The bottom and the top of each box are indicated. The 25th and 75th percentiles are represented by the ends of the whiskers, which indicate the minimum and maximum values of all data, respectively. *p < 0.05. (A–C) One‐way analysis anova with Tukey's test. irAE, immune‐related adverse event; n.s., not significant.

We further examined whether the eosinophil proportion in the two‐course sample was associated with the time of irAE occurrences. Of the 419 irAEs, 25.8% (108 events) occurred within the first 4 weeks of treatment, 32.4% (136 events) occurred between 4 and 12 weeks after treatment, and 38.9% (163 events) occurred at least 12 weeks after treatment (Table S3). Remarkably, the eosinophil proportion in the two‐course sample was substantially elevated by irAE occurrence compared with that in the non‐irAE group, regardless of when the irAEs occurred (0–2 weeks vs. non‐irAE group; mean, 5.0% vs. 2.5%, 2–4 weeks vs. non‐irAE group; mean, 5.0% vs. 2.5%, 4–12 weeks vs. non‐irAE group; mean, 4.8% vs. 2.5%, 12–24 weeks vs. non‐irAE group; mean, 3.7% vs. 2.5%, 24 weeks vs. non‐irAE group; mean, 4.1% vs. 2.5%; *p* < 0.05; Figure 3A).

### 3.6 Relationship between eosinophil proportion and irAE type/severity

Next, we examined whether the eosinophil proportion in the two‐course sample was associated with the development of any type of irAEs, including endocrine, gastrointestinal, skin, pulmonary, and other disorders. Patients in the irAE group who experienced endocrine (mean: 5.5% vs. 2.5%), gastrointestinal (mean: 4.2% vs. 2.5%), skin (mean: 4.7% vs. 2.5%), pulmonary (mean: 4.8% vs. 2.5%), or other disorders (mean: 5.1% vs. 2.5%) had a significantly higher eosinophil proportion in the two‐course sample than patients in the non‐irAE group (*p* < 0.05; Figure 3B). Analysis of each grade demonstrated that eosinophil proportion in the two‐course sample of patients who had experienced any grade irAEs significantly increased compared with those of non‐irAE group (grade 1 vs. non‐irAE group; mean: 3.7% vs. 2.5%, grade 2 vs. non‐irAE group; mean: 4.5% vs. 2.5%, grade ≥3 vs. non‐irAE group; mean: 4.6% vs. 2.5%; *p* < 0.05; Figure 3C). There was no difference among grade 1, grade 2, and grade ≥3 (grade 1 vs. grade 2; mean, 3.7% vs. 4.5%, grade 1 vs. grade ≥3; mean, 3.7% vs. 4.6%, grade 2 vs. grade ≥3; mean, 4.5% vs. 4.6%; Figure 3C).

### 3.7 Risk factor associated with irAE occurrences

The receiver operating characteristic (ROC) curve was used to identify the cutoff value of the eosinophil proportion for irAE occurrences. The optimum cutoff value of eosinophil proportion in the two‐course sample for irAE occurrences was 3.0% (area under the curve = 0.668, 95% CI = 0.62–0.71, sensitivity = 0.55, specificity = 0.68; Figure 3D). In the univariate logistic regression analysis of irAE occurrences, eosinophils of ≥3.0% in the two‐course sample were associated with high risk for irAEs occurrences (odds ratio (OR) = 2.74; 95% CI = 1.96–3.84; *p* < 0.05; Table 2). Moreover, multivariate logistic regression analyses demonstrated that eosinophil proportion was found to be an independent risk factor for the development of any grade irAEs (OR = 2.57; 95% CI = 1.79–3.67; *p* < 0.05; Table 2). Subgroup analysis showed that increased eosinophils of ≥3.0% have no heterogeneity in irAE occurrences (Figure S2).

**TABLE 2:** Univariate and multivariate logistic regression analyses of risk factors for the occurrence of irAEs.

	Univariate			Multivariate		
	OR	95% CI	*p* value	OR	95% CI	*p* value
Age: ≥65 years	1.01	0.68–1.48	0.97	1.11	0.73–1.69	0.62
Sex: male	1.35	0.94–1.93	0.10	1.43	0.95–2.15	0.08
Esophageal cancer: yes	0.71	0.34–1.46	0.35	1.38	0.36–5.21	0.63
Gastric cancer: yes	0.43	0.22–0.87	<0.05	0.98	0.26–3.62	0.97
Head and neck cancer: yes	1.23	0.78–1.95	0.36	2.42	0.73–7.93	0.14
Lung cancer: yes	0.77	0.53–1.12	0.17	1.22	0.39–3.79	0.73
Melanoma: yes	2.51	1.45–4.35	<0.05	4.39	1.27–15.2	<0.05
Renal cell carcinoma: yes	2.17	1.42–3.34	<0.05	2.56	0.72–9.09	0.14
Urothelial carcinoma: yes	0.51	0.33–0.78	<0.05	0.96	0.30–3.11	0.95
Anti‐PD‐1: yes	0.38	0.26–0.57	<0.05	0.38	0.19–0.76	<0.05
Anti‐PD‐L1: yes	1.62	0.89–2.95	0.11			
Anti‐CTLA‐4 plus anti‐PD‐1: yes	2.82	1.74–4.55	<0.05	0.61	0.22–1.70	0.35
Anti‐CTLA‐4: yes	8.74 × 10^4^	0‐inf	0.98			
Use of steroids before ICIs therapy: yes	2.25	0.40–12.4	0.35			
Proportion of eosinophils in two‐course sample: ≥3.0%	2.74	1.96–3.84	<0.05	2.57	1.79–3.67	<0.05

Abbreviations: CI, confidence interval; CTLA‐4, cytotoxic T‐lymphocyte antigen 4; irAEs, immune‐related adverse events; OR, odds ratio; PD‐1, Programmed death 1; PD‐L1, Programmed death ligand 1.

### 3.8 Eosinophil proportion is an effective biomarker to predict OS and PFS

**FIGURE 4:**
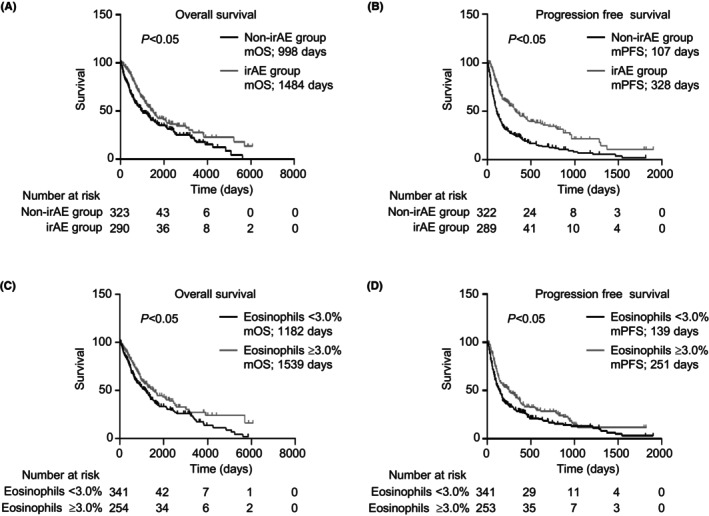
Elevation of eosinophil proportion associated with clinical outcome. (A–D) Kaplan–Meier survival curves for: (A) overall survival (non‐irAE group: *n* = 323; irAE group: *n* = 290); (B) progression‐free survival (non‐irAE group: *n* = 322; irAE group: *n* = 289); (C) overall survival (eosinophils <3.0%: *n* = 341; eosinophils ≥3.0%: *n* = 254); and (D) progression‐free survival (eosinophils <3.0%: *n* = 341; eosinophils ≥3.0%: *n* = 253) in patients. (A–D) Log‐rank test. irAE, immune‐related adverse event; mOS, median overall survival; mPFS, median progression‐free survival.

The median OS (mOS) and PFS (mPFS) of the irAE group were significantly longer than those of the non‐irAE group (mOS, *p* < 0.05; mPFS, *p* < 0.05; Figure 4A,B). Eosinophils of ≥3.0% in the two‐course sample correlated with better mOS and mPFS (mOS, *p* < 0.05; mPFS, *p* < 0.05; Figure 4C,D). Consistently, eosinophils of ≥3.0% in the two‐course sample were a factor for indicating better OS in both univariate and multivariate cox regression analyses (univariate cox regression analyses: hazard ratio (HR) = 0.71, 95% CI = 0.56–0.90; *p* < 0.05, multivariate cox regression analyses: HR = 0.77, 95% CI = 0.59–0.99, *p* < 0.05, Table S4).

### 3.9 Propensity score matching analysis between the irAE and non‐irAE groups

**FIGURE 5:**
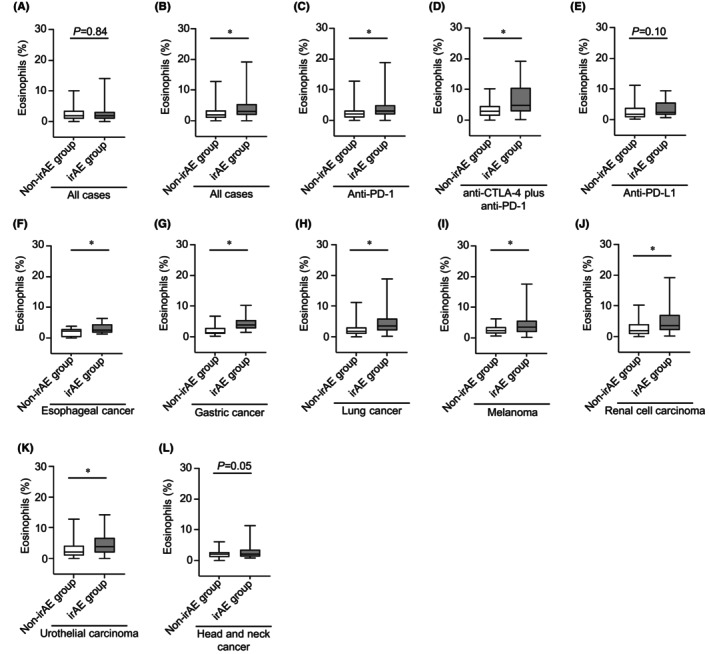
Analysis of the eosinophil proportion for each ICI and primary cancer type adjusted by propensity score matching. (A) Boxplot showing the eosinophil proportion in the baseline sample: non‐irAE (*n* = 210) and irAE (*n* = 216) groups. (B) Boxplot showing the eosinophil proportion in the two‐course sample; non–irAE (*n* = 206) and irAE (*n* = 214) groups. (C–E) Boxplot showing eosinophil proportion in a two‐course sample of patients who received (C) anti‐PD‐1 monotherapy (non‐irAE [*n* = 165] and irAE [*n* = 172] groups), (D) anti‐CTLA‐4 plus anti‐PD‐1 combination therapy (non‐irAE [*n* = 23] and irAE [*n* = 23] groups), and (E) anti‐PD‐L1 monotherapy (non‐irAE [*n* = 18] and irAE [*n* = 19] groups). (F–L) Boxplot showing eosinophil proportion in a two‐course sample of patients with (F) esophageal cancer (non‐irAE [*n* = 12] and irAE [*n* = 12] groups), (G) Gastric cancer (non‐irAE [*n* = 11] and irAE [*n* = 12] groups), (H) lung cancer (non‐irAE [*n* = 58] and irAE [*n* = 60] groups), (I) melanoma (non‐irAE [*n* = 19] and irAE [*n* = 22] groups), (J) renal cell carcinoma (non‐irAE [*n* = 35] and irAE [*n* = 38] groups), (K) urothelial carcinoma (non‐irAE [*n* = 32] and irAE [*n* = 34] groups), and (L) head and neck cancer (non‐irAE [*n* = 33] and irAE [*n* = 31] groups). The median value is represented by the horizontal midline in each box. The bottom and the top of each box are indicated. The 25th and 75th percentiles are represented by the ends of the whiskers, which indicate the minimum and maximum values of all data, respectively. *p < 0.05. (A–L) Unpaired *t* test. irAE, immune‐related adverse event; PD‐1, Programmed death 1; PD‐L1, Programmed death ligand 1.

**FIGURE 6:**
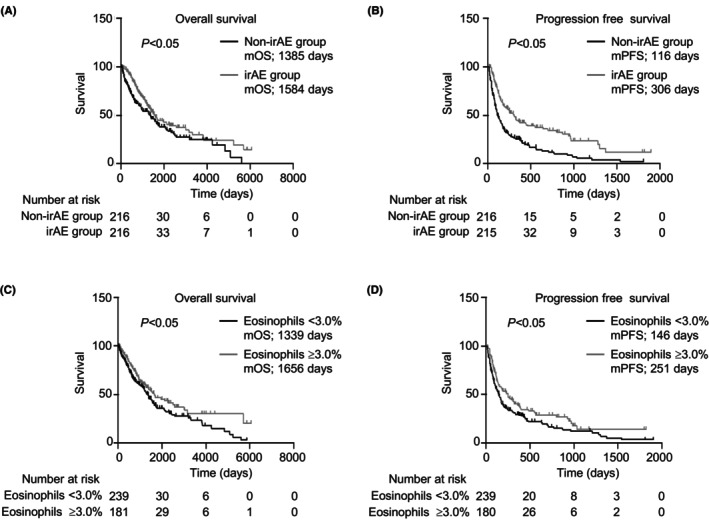
Analysis of mOS and mPFS after propensity score matching. (A–D) Kaplan–Meier survival curves for: (A) overall survival (non‐irAE group: *n* = 216; irAE group: *n* = 216), (B) progression‐free survival (non‐irAE group: *n* = 216; irAE group: *n* = 215), (C) overall survival (eosinophils <3.0%: *n* = 239; eosinophils ≥3.0%: *n* = 181), and (D) progression‐free survival (eosinophils <3.0%: *n* = 239; eosinophils ≥3.0%: *n* = 180) in patients. (A–D) Log‐rank test. irAE, immune‐related adverse event; mOS, median overall survival; mPFS, median progression‐free survival.

The patients' characteristics of the two groups after matching are listed in Table 3. We also examined the variation of eosinophils and efficacy after propensity score matching. Consistent with the results before propensity score matching, the eosinophil proportion and count in two‐course sample of all cases was significantly higher in the irAE group than in the non‐irAE group, but not the baseline sample (Figure 5A,B and Figure S3). Furthermore, in the analysis of each type of ICIs and primary tumor, the eosinophils in the irAE group in the two‐course sample were substantially higher than those in the non‐irAE group regardless of type of ICIs and primary tumor (Figure 5C–L). The irAE group and eosinophils of ≥3.0% in the two‐course sample were significantly correlated with better mOS and mPFS, consistent with the results before propensity score matching (mOS, *p* < 0.05; mPFS, *p* < 0.05; Figure 6A–D).

**TABLE 3:** Clinical features of patients adjusted by propensity score matching.

Characteristics	Non‐irAE group	irAE group	*p* value
Total, *n* (%)	216	216	
Age (range)	74 (32–92)	74 (41–90)	0.64
Sex, *n* (%)			
Male	176 (81.5)	177 (81.9)	1.00
Female	40 (18.5)	39 (18.1)	
Primary tumor type, *n* (%)			
Esophageal cancer	13 (6.00)	13 (6.00)	1.00
Gastric cancer	12 (5.60)	12 (5.60)	1.00
Head and neck cancer	33 (15.3)	31 (14.4)	0.89
Lung cancer	60 (27.8)	60 (27.8)	1.00
Melanoma	20 (9.30)	23 (10.6)	0.74
Renal cell carcinoma	40 (18.5)	38 (17.6)	0.90
Urothelial carcinoma	33 (15.3)	34 (15.7)	1.00
Other cancers	5 (2.30)	5 (2.30)	1.00
Colorectal cancer	1	1	
Cancer of unknown primary	0	2	
Mediastinal tumor	1	0	
Merkel cell carcinoma	2	2	
Mesothelioma	1	0	
Immune checkpoint therapy, *n* (%)			
Anti‐PD‐1	171 (79.2)	174 (80.6)	0.81
Nivolumab	95	103	
Pembrolizumab	76	71	
Anti‐PD‐L1	18 (8.30)	19 (8.80)	1.00
Atezolizumab	11	4	
Avelumab	2	3	
Durvalumab	5	12	
Anti‐CTLA‐4 plus anti‐PD‐1	27 (12.5)	23 (10.6)	0.65
Anti‐CTLA‐4	0 (0.0)	0 (0.0)	NA
Use of steroids before ICIs, n (%)	2 (0.9)	3 (1.4)	1.00

Abbreviations: CTLA‐4, cytotoxic T‐lymphocyte antigen 4; ICIs, immune checkpoint inhibitors; irAE, immune‐related adverse event; NA, not applicable; PD‐1, Programmed death 1; PD‐L1, Programmed death ligand 1.

## 4 DISCUSSION

In the current study, we clarified that an elevated eosinophil proportion before two‐course treatment may be a predictor of irAE occurrences common to patients treated with different ICIs. Consistent with our data (Table S2), systematic review revealed that OR of grade ≥3 irAEs in anti‐PD‐1 monotherapy is 1.58‐fold higher than in anti‐PD‐L1 monotherapy.^14^ Our study and previous studies collectively indicate that differences in the incidence of irAEs may contribute to ICI type. However, to date, no predictor of irAE common to different types of ICI has been reported, as most studies have focused on specific ICI types. We found that the proportion of eosinophils before the two‐course treatment was elevated in the irAE group treated with any ICIs. Our approach using the eosinophil proportion before the two‐course treatment may provide a new strategy for the prediction of irAEs.

A previous study reported that the incidence of irAEs did not differ among the cancer types.^14^ Contrarily, the current study showed the different incidents of irAEs among cancer type treated with anti‐PD‐1 monotherapy such as the highest incidences of irAEs was 62.3% in melanoma, and the lowest incidence of irAEs was 29.3% in gastric cancer (Table S1). Additionally, multivariate logistic regression analyses demonstrated that melanoma was higher odds ratio (OR = 4.39), and anti‐PD‐1 monotherapy was lower odds ratio (OR = 0.38; Table 2). Our data indicated that incidents of irAEs might be different among cancer type. The efficacy of ICIs has been reported to be related to irAEs occurrences and tumor mutational burden (TMB).^16^, ^24^, ^25^, ^26^, ^27^, ^28^, ^29^ Melanoma have the highest rate of TMB ≥ 10 mutation/Mb.^28^ The urothelial carcinoma and gastrointestinal cancer treated with anti‐PD‐1 monotherapy have lower TMB ≥ 10 mutation/M than melanoma, lung cancer, and head and neck cancer.^28^ Although it is unclear whether irAE occurrence is related to TMB, our data may explain their association. We found that an increased eosinophil proportion reflected irAE occurrence in various primary tumor types, regardless of the incidence of irAEs. Therefore, using eosinophil as a predictor of irAE occurrence may help physicians in many specialty areas. As these controversial results between our data and those of other study groups may be reflected in different patient characteristics, such as types of cancers and ICIs, our results should be validated in a larger external cohort.

Previous studies have reported that irAEs occur not only in the early phase of ICI treatment, but also in the late phase of treatment.^30^ In the irAE profile of melanoma patients treated with anti‐PD‐1 monotherapy, approximately 80% of the irAEs occurred within 16 weeks of the first treatment.^31^ Skin and gastrointestinal disorders are observed from 5 to 7 weeks and tend to increase approximately 10–20 weeks after the start of treatment. Endocrine and pulmonary disorders were observed from 7 to 10 weeks and tended to increase in incidence until 30 weeks after the start of treatment.^31^ The onset of skin and gastrointestinal disorders induced by anti‐CTLA‐4 monotherapy tends to be earlier than that induced by anti‐PD‐1 monotherapy, such as within the first 3–5 weeks of treatment.^32^ In addition, irAEs may occur up to 6 months after discontinuation of ICIs,^33^ and 28.8% of ICI‐rechallenged patients experience the same irAEs associated with the discontinuation of ICIs.^30^ Consistent with previous studies, our data showed that 65.1% of patients experienced irAEs of any grade within 16 weeks of the first treatment (Table S3). Surprisingly, 21.5% of patients experienced irAEs 24 weeks or more after treatment (Table S3). Previous studies and our data have demonstrated that it is difficult to identify when irAEs occur, as irAEs may occur early to late with ICIs and even after their completion. Our study showed that an increased eosinophil proportion before two courses of ICIs could predict irAE occurrence, regardless of when the irAEs occurs and the irAE type/severity. Therefore, we suggest the necessity of examining eosinophils before two courses of ICI therapy to predict the occurrence of irAEs.

Increased eosinophils induced by ICIs have been associated with prognostic biomarkers for ICIs in various cancers.^27^ Mechanistically, microarray analysis using RNA isolated from eosinophils under ICI treatment in melanoma revealed that Wnt signaling prolongs the survival of activated eosinophils.^34^ Activated eosinophils accumulate in tumor tissues, indicating that tumor‐infiltrating eosinophils eliminate cancer cells via vascular normalization and macrophage polarization.^35^, ^36^, ^37^ Thus, despite reports on the functional analysis of eosinophils under ICI treatment, the detailed underlying mechanisms by which eosinophils are increased in peripheral blood and infiltrate into tumors have not been fully clarified. In that situation, the latest studies have clarified a part of these mechanisms.^38^ Blomberg et al. reported that interleukin‐5 secreted by CD4^+^ T cells after ICI treatment increases eosinophil production in the bone marrow and accumulates eosinophils in the peripheral blood of patients with breast cancer. Moreover, increased eosinophil infiltration into tumors by interleukin 33 exerts its antitumor effect by activating CD8^+^ T cells.^38^ These mechanisms may explain why eosinophils are increased after ICI. In addition, as several studies have shown, the infiltration of eosinophils accumulated by ICI into normal tissues signifies irAE occurrences.^39^, ^40^, ^41^, ^42^ Therefore, several studies have shown that an increase in eosinophil is a biomarker of irAEs occurences.^17^, ^18^, ^19^, ^20^ Although the detailed underlying mechanisms about association between eosinophils and irAEs occurrence are unclear, previous studies supported our data that increased eosinophils may reflect efficacy and safety in patients treated with ICIs.^16^, ^18^, ^24^, ^25^, ^26^, ^27^, ^38^

In the past, most studies have shown that ICI‐mediated irAEs may be associated with an improved response to therapy and survival outcomes in several types of cancers.^43^, ^44^, ^45^, ^46^ Consistently, our data revealed significantly longer mOS and mPFS in patients with irAEs than in those without irAEs, as reported in another study (Figure 4A,B). In contrast, meta‐analysis showed irAE occurrences of grade <2 associated with better OS, but not grade ≥3.^47^, ^48^ In addition, in a study of 42‐month results of the CheckMate 214 trial, treatment‐free survival of patients who experienced an irAE of grade ≥3 tended to be shorter than that of patients who did not experience an irAE of grade ≥3 (0.6 and 6.1 months, respectively).^49^ The American Society of Clinical Oncology Clinical Practice guidelines recommend the administration of corticosteroids and the discontinuation of ICIs, depending on the type and grade of irAEs.^50^ However, these studies on the association between prognosis and irAEs indicated that physicians should not discontinue treatment, but rather should diagnose irAEs early and control irAEs below grade 2. In this study, we discovered that increasing eosinophils of ≥3.0% before two‐course treatment may be a practical biomarker of irAE occurrences. Therefore, physicians should carefully evaluate the eosinophil proportion for early prediction and management of irAEs to prevent severe irAEs.

The present study had some limitations. Owing to the retrospective nature of the study, we could not control for bias in patient selection. However, a prospective interventional study is required to confirm these findings.

In conclusion, the present study showed that increased eosinophils play a crucial role in predicting irAE occurrence, regardless of the primary tumor, ICIs, or irAE type. Our data highlight the importance of measuring eosinophils to predict irAE occurrences before the two‐course ICI treatment.

## AUTHOR CONTRIBUTIONS

**Yoshihiko Tasaki:** Conceptualization (lead); data curation (lead); formal analysis (lead); funding acquisition (lead); investigation (lead); methodology (lead); project administration (lead); resources (lead); software (lead); validation (equal); visualization (equal); writing – original draft (lead); writing – review and editing (lead). **Yosuke Sugiyama:** Conceptualization (supporting); formal analysis (equal); methodology (equal); resources (supporting); software (supporting); supervision (supporting); writing – original draft (supporting); writing – review and editing (supporting). **Shuzo Hamamoto:** Conceptualization (supporting); data curation (supporting); resources (supporting); supervision (supporting); writing – original draft (supporting); writing – review and editing (supporting). **Taku Naiki:** Conceptualization (supporting); data curation (supporting); resources (supporting); supervision (supporting); writing – original draft (supporting); writing – review and editing (supporting). **Takehiro Uemura:** Data curation (supporting); resources (supporting); supervision (supporting); writing – original draft (supporting); writing – review and editing (supporting). **Keisuke Yokota:** Data curation (supporting); resources (supporting); supervision (supporting); writing – original draft (supporting); writing – review and editing (supporting). **Daisuke Kawakita:** Data curation (supporting); resources (supporting); supervision (supporting); writing – original draft (supporting); writing – review and editing (supporting). **Motoki Nakamura:** Data curation (supporting); resources (supporting); supervision (supporting); writing – original draft (supporting); writing – review and editing (supporting). **Ryo Ogawa:** Data curation (supporting); resources (supporting); supervision (supporting); writing – original draft (supporting); writing – review and editing (supporting). **Takaya Shimura:** Data curation (supporting); resources (supporting); supervision (supporting); writing – original draft (supporting); writing – review and editing (supporting). **Yoshihisa Mimura:** Conceptualization (supporting); data curation (supporting); resources (supporting); supervision (supporting); writing – original draft (supporting); writing – review and editing (supporting). **Yuji HOTTA:** Data curation (supporting); resources (supporting); supervision (supporting); writing – original draft (supporting); writing – review and editing (supporting). **Kunihiro Odagiri:** Data curation (supporting); resources (supporting); supervision (supporting); writing – original draft (supporting); writing – review and editing (supporting). **Nanami Ito:** Data curation (supporting); resources (supporting); supervision (supporting); writing – original draft (supporting); writing – review and editing (supporting). **Moeko Iida:** Data curation (supporting); resources (supporting); supervision (supporting); writing – original draft (supporting); writing – review and editing (supporting). **Yuka Kimura:** Data curation (supporting); resources (supporting); supervision (supporting); writing – original draft (supporting); writing – review and editing (supporting). **Hirokazu Komatsu:** Data curation (supporting); resources (supporting); supervision (supporting); writing – original draft (supporting); writing – review and editing (supporting). **Hiromi Kataoka:** Data curation (supporting); resources (supporting); supervision (supporting); writing – original draft (supporting); writing – review and editing (supporting). **Shuji Takiguchi:** Data curation (supporting); resources (supporting); supervision (supporting); writing – original draft (supporting); writing – review and editing (supporting). **Akimichi Morita:** Data curation (supporting); resources (supporting); supervision (supporting); writing – original draft (supporting); writing – review and editing (supporting). **Shinichi Iwasaki:** Data curation (supporting); resources (supporting); supervision (supporting); writing – original draft (supporting); writing – review and editing (supporting). **Katsuhiro Okuda:** Data curation (supporting); resources (supporting); supervision (supporting); writing – original draft (supporting); writing – review and editing (supporting). **Akio Niimi:** Data curation (supporting); resources (supporting); supervision (supporting); writing – original draft (supporting); writing – review and editing (supporting). **Takahiro Yasui:** Conceptualization (supporting); data curation (supporting); resources (supporting); supervision (supporting); writing – original draft (supporting); writing – review and editing (supporting). **Yoko Furukawa#x2010;Hibi:** Conceptualization (supporting); data curation (supporting); funding acquisition (supporting); resources (supporting); supervision (supporting); writing – original draft (supporting); writing – review and editing (supporting).

## FUNDING INFORMATION

This study was conducted as a part of the research program of the Nitto Foundation (Y.T.).

## CONFLICT OF INTEREST STATEMENT

The authors declare no conflict of interest.

## ETHICS APPROVAL STATEMENT

This study was conducted in accordance with the Declaration of Helsinki and approved by the Institutional Review Board of Nagoya City University Graduate School of Medical Science (approval number 60‐22‐0076).

## PATIENT CONSENT STATEMENT

For retrospective study, the need for consent to participate was deemed unnecessary according to national regulations, “Ethical Guidelines for Medical and Health Research Involving Human Subjects.” The opportunity to opt out was provided to patients on the hospital website.

## Supporting information


Figure S1‐S3.

Table S1‐S4.

## Data Availability

The author confirms that the data of this study are available within the manuscript and its supplementary figures and tables.
