# Supplementary material for: Eosinophil may be a predictor of immune‐related adverse events induced by different immune checkpoint inhibitor types: A retrospective multidisciplinary study
Source: Cancer Med. 2023 Nov 21;12(24):21666–79. doi: 10.1002/cam4.6724 (PMC10757154; doi:10.1002/cam4.6724)
Supplement: Supplementary file 1 — Figure S1‐S3. [file CAM4-12-21666-s002.pptx]

## Slide 1
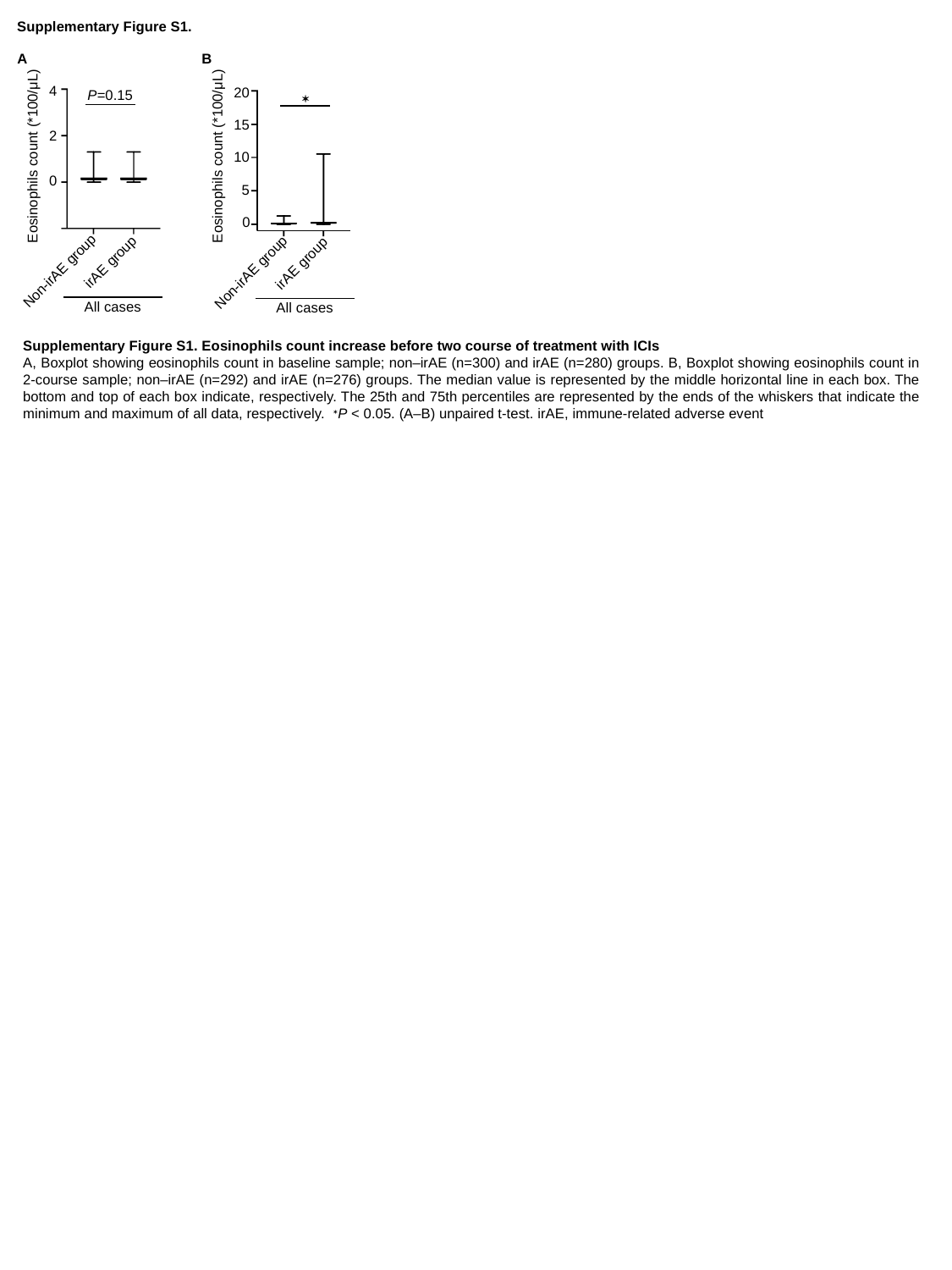

Supplementary Figure S1.
A
B
P=0.15
4
*
20
15
2
Eosinophils count (*100/μL)
Eosinophils count (*100/μL)
10
0
5
0
irAE group
irAE group
Non-irAE group
Non-irAE group
All cases
All cases
Supplementary Figure S1. Eosinophils count increase before two course of treatment with ICIs
A, Boxplot showing eosinophils count in baseline sample; non–irAE (n=300) and irAE (n=280) groups. B, Boxplot showing eosinophils count in 2-course sample; non–irAE (n=292) and irAE (n=276) groups. The median value is represented by the middle horizontal line in each box. The bottom and top of each box indicate, respectively. The 25th and 75th percentiles are represented by the ends of the whiskers that indicate the minimum and maximum of all data, respectively. P < 0.05. (A–B) unpaired t-test. irAE, immune-related adverse event

## Slide 2
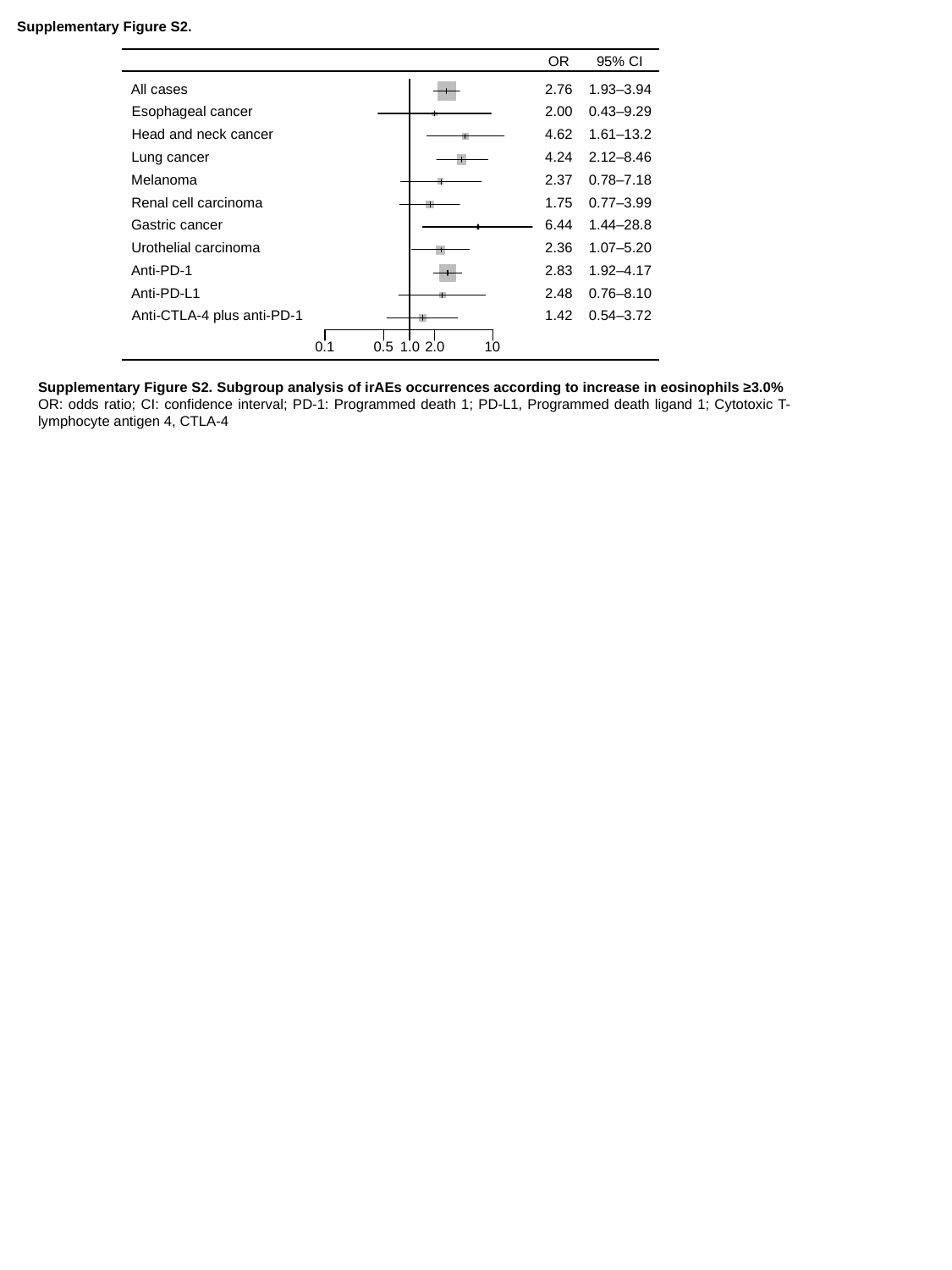

OR
95% CI
All cases
2.76
1.93–3.94
Esophageal cancer
2.00
0.43–9.29
Head and neck cancer
4.62
1.61–13.2
Lung cancer
4.24
2.12–8.46
Melanoma
2.37
0.78–7.18
Renal cell carcinoma
1.75
0.77–3.99
Gastric cancer
6.44
1.44–28.8
Urothelial carcinoma
2.36
1.07–5.20
Anti-PD-1
2.83
1.92–4.17
Anti-PD-L1
2.48
0.76–8.10
Anti-CTLA-4 plus anti-PD-1
1.42
0.54–3.72
0.1
0.5
1.0
2.0
10
Supplementary Figure S2.
Supplementary Figure S2. Subgroup analysis of irAEs occurrences according to increase in eosinophils ≥3.0%
OR: odds ratio; CI: confidence interval; PD-1: Programmed death 1; PD-L1, Programmed death ligand 1; Cytotoxic T-lymphocyte antigen 4, CTLA-4

## Slide 3
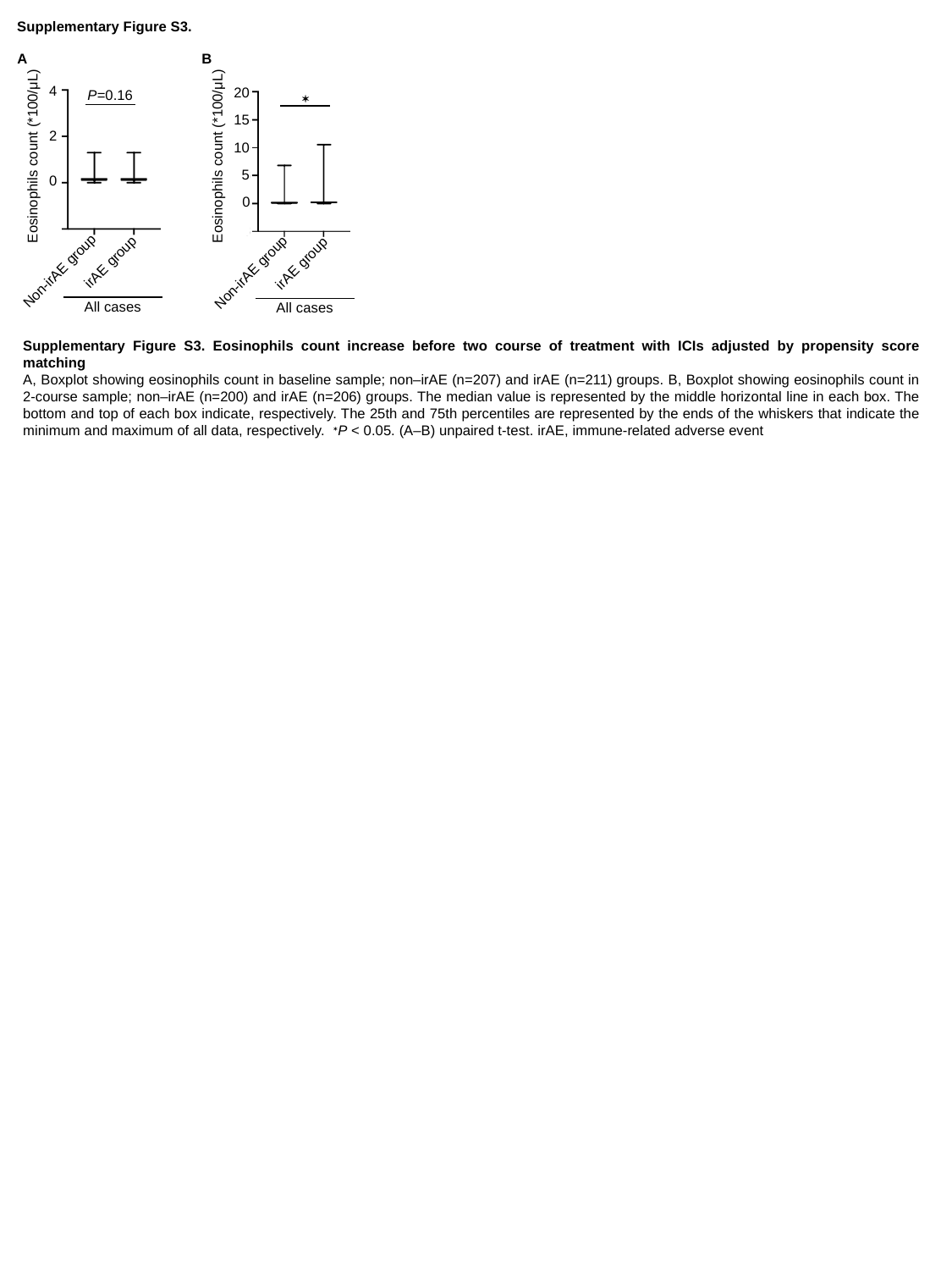

Supplementary Figure S3.
A
B
P=0.16
4
*
20
15
2
10
Eosinophils count (*100/μL)
Eosinophils count (*100/μL)
5
0
0
irAE group
irAE group
Non-irAE group
Non-irAE group
All cases
All cases
Supplementary Figure S3. Eosinophils count increase before two course of treatment with ICIs adjusted by propensity score matching
A, Boxplot showing eosinophils count in baseline sample; non–irAE (n=207) and irAE (n=211) groups. B, Boxplot showing eosinophils count in 2-course sample; non–irAE (n=200) and irAE (n=206) groups. The median value is represented by the middle horizontal line in each box. The bottom and top of each box indicate, respectively. The 25th and 75th percentiles are represented by the ends of the whiskers that indicate the minimum and maximum of all data, respectively. P < 0.05. (A–B) unpaired t-test. irAE, immune-related adverse event
