# Supplementary material for: Eosinophil may be a predictor of immune‐related adverse events induced by different immune checkpoint inhibitor types: A retrospective multidisciplinary study
Source: Cancer Med. 2023 Nov 21;12(24):21666–79. doi: 10.1002/cam4.6724 (PMC10757154; doi:10.1002/cam4.6724)
Supplement: Supplementary file 2 — Table S1‐S4. [file CAM4-12-21666-s001.docx]

**Supplementary Table 1. Incidence of irAEs in various cancer patients treated each ICIs**

|  | Anti-PD-1 | | | Anti-PD-L1 | | | Anti-CTLA-4 plus anti-PD-1 | | |
| --- | --- | --- | --- | --- | --- | --- | --- | --- | --- |
| Group | All cases | Non-irAE group | irAE group | All cases | Non-irAE group | irAE group | All cases | Non-irAE group | irAE group |
| Total, n (%) | 476 (100) | 276 (58.0) | 200 (42.0) | 48 (100) | 20 (41.7) | 28 (58.3) | 89 (100) | 28 (31.5) | 61 (68.5) |
| Esophageal cancer | 33 (100) | 20 (60.6) | 13 (39.4) | - | - | - | - | - | - |
| Gastric cancer | 41 (100) | 29 (71.7) | 12 (29.3) | - | - | - | - | - | - |
| Head and neck cancer | 85 (100) | 41 (48.2) | 44 (51.8) | - | - | - | - | - | - |
| Lung cancer | 113 (100) | 69 (61.1) | 44 (38.9) | 37 (100) | 17 (45.9) | 20 (54.1) | 1 (100) | 1 (100) | 0 (0.0) |
| Melanoma | 53 (100) | 20 (37.7) | 33 (62.3) | - | - | - | 10 (100) | 1 (10.0) | 9 (90.0) |
| Renal cell carcinoma | 31 (100) | 14 (45.2) | 17 (54.8) | - | - | - | 77 (100) | 26 (33.8) | 51 (66.2) |
| Urothelial carcinoma | 110 (100) | 76 (69.1) | 34 (30.9) | 7 (100) | 1 (14.3) | 6 (85.7) | - | - | - |
| Other cancers | 10 (100) | 7 (70.0) | 3 (30.0) | 4 (100) | 2 (50.0) | 2 (50.0) | 1 (100) | 0 (0.0) | 1 (100) |

irAE: immune-related adverse event; PD-1: Programmed death 1; PD-L1, Programmed death ligand 1; Cytotoxic T-lymphocyte antigen 4, CTLA-4; ICIs: immune checkpoint inhibitors

**Supplementary Table 2. Profile of irAEs**

|  | All cases | | Anti-PD-1 | | Anti-PD-L1 | | Anti-CTLA-4 plus anti-PD-1 | | Anti-CTLA-4 | |
| --- | --- | --- | --- | --- | --- | --- | --- | --- | --- | --- |
| Profile of irAEs | All grade | ≥Grade 3 | All grade | ≥Grade 3 | All grade | ≥Grade 3 | All grade | ≥Grade 3 | All grade | ≥Grade 3 |
| All events, n (%) | 419 (100) | 111 (26.5) | 276 (65.9) | 65 (23.6) | 40 (9.6) | 5 (12.5) | 102 (24.3) | 41 (40.2) | 1 (0.2) | 0 (0.0) |
| Skin | 122 (29.1) | 8 | 86 | 7 | 17 | 0 | 19 | 1 | 0 | 0 |
| Endocrine | 92 (22.0) | 26 | 67 | 16 | 3 | 0 | 21 | 10 | 1 | 0 |
| Gastrointestinal | 91 (21.7) | 40 | 60 | 24 | 6 | 2 | 25 | 14 | 0 | 0 |
| Pulmonary | 45 (10.7) | 16 | 27 | 7 | 6 | 2 | 12 | 7 | 0 | 0 |
| Other disorders | 69 (16.5) | 21 | 36 | 11 | 8 | 1 | 25 | 9 | 0 | 0 |

irAEs: immune-related adverse events; PD-1: Programmed death 1; PD-L1, Programmed death ligand 1; Cytotoxic T-lymphocyte antigen 4, CTLA-4

**Supplementary Table 3. Occurrence of irAEs of any grade over time**

|  |  | Time from initiation of ICIs to irAEs occurrences (weeks) | | | | | | | | |
| --- | --- | --- | --- | --- | --- | --- | --- | --- | --- | --- |
|  |  | 0~2 | 2~4 | 4~8 | 8~12 | 12~16 | 16~20 | 20~24 | 24~ | Not evaluated |
| All events, n (%) | 419 (100) | 48 (11.5) | 60 (14.3) | 81 (19.3) | 55 (13.1) | 29 (6.9) | 31 (7.4) | 13 (3.1) | 90 (21.5) | 12 (2.9) |
| Skin | 122 (29.1) | 13 (3.1) | 23 (5.5) | 27 (6.4) | 13 (3.1) | 6 (1.4) | 6 (1.4) | 4 (1.0) | 24 (5.7) | 3 (0.7) |
| Endocrine | 92 (22.0) | 3 (0.7) | 13 (3.1) | 22 (5.3) | 14 (3.3) | 9 (2.1) | 11 (2.6) | 3 (0.7) | 20 (4.8) | 2 (0.5) |
| Gastrointestinal | 91 (21.7) | 12 (2.9) | 10 (2.4) | 14 (3.3) | 14 (3.3) | 6 (1.4) | 10 (2.4) | 4 (1.0) | 19 (4.5) | 3 (0.7) |
| Pulmonary | 45 (10.7) | 2 (0.5) | 6 (1.4) | 7 (1.7) | 8 (1.9) | 6 (1.4) | 1 (0.2) | 1 (0.2) | 12 (2.9) | 0 (0.0) |
| Other disorders | 69 (16.5) | 18 (4.3) | 8 (1.9) | 11 (2.6) | 6 (1.4) | 2 (0.5) | 3 (0.7) | 1 (0.2) | 15 (3.6) | 4 (1.0) |

irAEs: immune-related adverse events; ICIs: immune checkpoint inhibitors

**Supplementary Table 4. Univariate and multivariate cox regression analysis of risk factors for predicting overall survival**

|  | Univariate | | |  | Multivariate | | |
| --- | --- | --- | --- | --- | --- | --- | --- |
|  | HR | 95%CI | *P* value |  | HR | 95%CI | *P* value |
| Age: ≥65 years | 0.98 | 0.74-1.29 | 0.91 |  | 1.03 | 0.77-1.37 | 0.81 |
| Esophageal cancer: Yes | 1.41 | 0.88-2.24 | 0.14 |  | 3.35 | 1.13-9.91 | <0.05 |
| Gastric cancer: Yes | 1.56 | 1.07-2.28 | <0.05 |  | 3.05 | 1.07-8.74 | <0.05 |
| Head and neck cancer: Yes | 1.05 | 0.76-1.45 | 0.73 |  | 2.04 | 0.72-5.73 | 0.17 |
| Lung cancer: Yes | 0.90 | 0.70-1.16 | 0.43 |  | 1.91 | 0.69-5.24 | 0.20 |
| Melanoma: Yes | 0.75 | 0.49-1.14 | 0.18 |  | 1.55 | 0.53-4.54 | 0.42 |
| Renal cell carcinoma: Yes | 0.60 | 0.42-0.85 | <0.05 |  | 1.05 | 0.36-3.05 | 0.91 |
| Urothelial carcinoma: Yes | 1.59 | 1.19-2.12 | <0.05 |  | 3.13 | 1.12-8.72 | <0.05 |
| Anti-PD-1 antibody: Yes | 1.05 | 0.77-1.43 | 0.73 |  | 1.15 | 0.70-1.89 | 0.56 |
| Anti-CTLA-4 plus anti-PD-1: Yes | 1.08 | 0.74-1.59 | 0.66 |  | 3.03 | 1.49-6.18 | <0.05 |
| Onset to irAEs: Yes | 0.63 | 0.50-0.80 | <0.05 |  | 0.66 | 0.51-0.85 | <0.05 |
| Proportion of eosinophils  in 2-course sample: ≥3.0% | 0.71 | 0.56-0.90 | <0.05 |  | 0.77 | 0.59-0.99 | <0.05 |

HR: hazard ratio; CI: confidence interval; PD-1: Programmed death 1; Cytotoxic T-lymphocyte antigen 4, CTLA-4; irAEs: immune-related adverse events;
